# Novel Mutations in the TMPRSS3 Gene May Contribute to Taiwanese Patients with Nonsyndromic Hearing Loss

**DOI:** 10.3390/ijms21072382

**Published:** 2020-03-30

**Authors:** Swee-Hee Wong, Yung-Chang Yen, Shuan-Yow Li, Jiann-Jou Yang

**Affiliations:** 1 Institute of Medicine, Chung Shan Medical University, Taichung 402, Taiwan; a7710355@gmail.com; 2 Department of BioMedical Sciences, Chung Shan Medical University, Taichung 402, Taiwan; 3 Department of Ophthalmology, Chi-Mei Medical Center, Liou-Ying, Tainan 736, Taiwan; dy5101@yahoo.com.tw; 4 Department of Nursing, Min Hwei College of Healthe Care Management, Tainan 736, Taiwan; 5 Department of Medical Research, Chung Shan Medical University Hospital, Taichung 402, Taiwan

**Keywords:** TMPRSS3, mutation, hearing loss, sGASP, xenopus oocyte

## Abstract

A previous study indicated that mutations in the transmembrane protease serine 3 (TMPRSS3) gene, which encodes a transmembrane serine protease, cause nonsyndromic hearing loss (NSHL). This was the first description of a serine protease involved in hearing loss (HL). In Taiwan, however, data on the TMPRSS3 gene’s association with NSHL is still insufficient. In this study, we described 10 mutations of TMPRSS3 genes found in 14 patients after screening 230 children with NSHL. The prevalence of the TMPRSS3 mutation appeared to be 6.09% (14/230). Of the 10 mutations, three were missense mutations: c.239G>A (p.R80H), c.551T>C (p.L184S), and 1253C>T (p.A418V); three were silent mutations, and four were mutations in introns. To determine the functional importance of TMPRSS3 mutations, we constructed plasmids carrying TMPRSS3 mutations of p.R80H, p.L184S, and p.A418V. TMPRSS3 function can be examined by secretory genetic assay for site-specific proteolysis (sGASP) and Xenopus oocyte expression system. Our results showed that p.R80H, p.L184S, and p.A418V TMPRSS3 mutations gave ratios of 19.4%, 13.2%, and 27.6%, respectively, via the sGASP system. Moreover, these three TMPRSS3 mutations failed to activate the epithelial sodium channel (ENaC) in the Xenopus oocyte expression system. These results indicate that the p.R80H, p.L184S, and p.A418V missense mutations of TMPRSS3 resulted in greatly diminishing the proteolytic activity of TMPRSS3. Our study provides information for understanding the importance of TMPRSS3 in the NSHL of Taiwanese children and provides a novel molecular explanation for the role of TMPRSS3 in HL.

## 1. Introduction

Hearing loss (HL) is a common sensory disorder with an incidence of about 1 child in 1000 births in the human population [1]. It is often caused by genetic inheritance of auditory system dysfunction; approximately 60% of HL is attributable to genetic factors [2,3]. Nearly 70% of genetic HL cases are nonsyndromic [3]. Recently, a whole exome sequencing (WES) study indicated that 64.3% (18/28) of patients with severe-to-profound congenital nonsyndromic hearing loss (NSHL) were determined to be genetically predisposed and were found to mostly exhibit an autosomal recessive (AR) inheritance pattern in these patients [4]. A total of 112 genes have been identified and described, some of which are involved in ion recycling and maintenance [5].

Transmembrane protease serine 3 (TMPRSS3) is part of the type II transmembrane serine proteases subfamily [6]. The *TMPRSS3* gene (OMIM 605511) contains 13 exons and spans approximately 24 kb at the chromosome 21q22.3 [7]. TMPRSS3′s encoding protein is an enzyme; its structure includes a short N-terminal tail, a transmembrane (TM) domain, and a large C-terminal segment. The large C-terminal encompasses a stem region within the low density lipoprotein receptor class A (LDLRA) and scavenger receptor cysteine rich (SRCR) domains, a potential proteolytic activation cleavage site between p.R216 and p.I217, and a serine protease domain containing the catalytic triad signature (p.H257, p.D304, and p.S401). This structure suggests that TMPRSS3 could be active as a membrane-bound enzyme [8].

A study on TMPRSS3 was the first to report that an enzyme can be associated with NSHL and that it can be related to maintaining Na^+^ within the endolymphatic environment in the cochlea [9,10]. To date, 31 different *TMPRSS3* mutations that lie in all functional domains have been described and have been reported to disrupt the proteolytic activity of TMPRSS3 in more than 14 ethnic groups worldwide, including Asian, Mediterranean, and Caucasian populations [11,12,13]. However, in Taiwan, data on the *TMPRSS3* gene associated with NSHL are still insufficient. Therefore, we aimed to determine the mutations of the *TMPRSS3* gene using comparative genetic analysis between genomic DNA from normal individuals and hearing-impaired patients in Taiwan. In addition, we studied the functional consequences of TMPRSS3 mutations by secretory genetic assay utilizing site-specific proteolysis (sGASP) and Xenopus oocyte expression systems. In this study, we found that 14 patients had variants in the *TMPRSS3* gene after screening 230 children with NSHL (14/230; 6.09%). By studying the function of TMPRSS3 missense mutations in HL, we found that the TMPRSS3 mutants causing HL fail to undergo proteolytic processing and to activate the epithelial sodium channel (ENaC) in vitro.

## 2. Results

### 2.1. TMPRSS3 Gene Screen in 230 Patients with NSHL

This study surveyed variants of the *TMPRSS3* gene in 350 unrelated individuals, 230 with NSHL and 120 with normal hearing. Seventeen variants of the *TMPRSS3* gene were found in both the patient and control groups, and were classified as a polymorphic site. The allelic and genotypic frequencies of polymorphism are summarized in Table 1. We made statistical chi-square test comparisons of the allelic frequencies on the polymorphic sites found in the patients and controls. The allele frequencies of most polymorphisms between the patients and controls did not differ significantly (*p* > 0.05), except for those of c.157G>A (p.V53I), c.447-13A>G, c.453G>A (p.V151V), c.617-4_617-5insAT, c.757A.>G (p.I253V), and c.1367G>A (Table 1).

**Figure 1:**
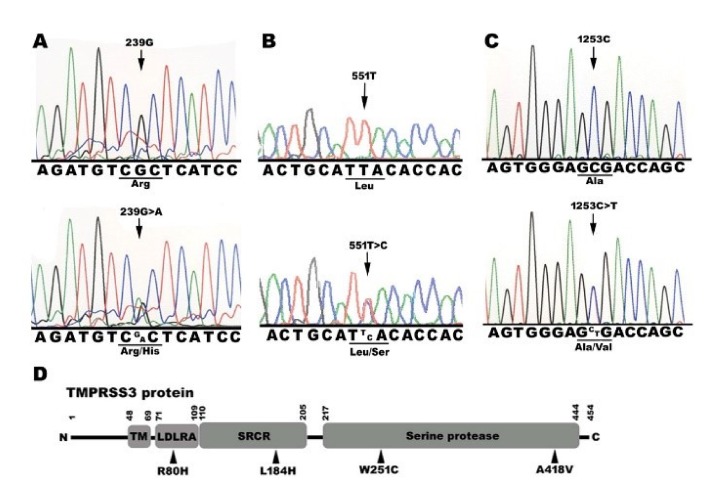
Sequence analysis of *TMPRSS3* genomic DNA variants in 230 patients with nonsyndromic hearing loss. Three variants of the *TMPRSS3* gene were found in this study, three heterozygous missence variants: (**A**) c.239G>A, (**B**) c.551T>C, and (**C**) c.1253C>T. (**D**) Schematic representation of the domain structure of the TMPRSS3 protein with indication of the variants (black triangle). N: N-terminal domain; TM: transmembrane domains; LDLAR: low density lipoprotein receptor class A; SRCR: scavenger receptor cysteine rich; C: C-terminal domain.

In addition, three categories of the *TMPRSS3* gene mutations carried by the patients with HL were found. They are shown in Table 2. The prevalence of *TMPRSS3* mutations appeared to be 6.09% (14/230). According to amino acid changes based on changes in nucleotides, the 10 mutations can be divided into three types. First, three were heterozygous missense mutations of c.239G>A (p.R80H) (Figure 1A), c.551T>C (p.L184S) (Figure 1B), and c.1253C>T (p.A418V) (Figure 1C). Second, three were heterozygous silent mutations of c.621T>C (p.C207C), c.933C>T (p.A311A), and c.1269C>T(p.I423I). Finally, there were four heterozygous mutations found in introns, including c.-75 A>G, c.205+38 C>T, c.1194+15 C>A, and c.1347+11 C>T. All of the variants were repeated two times using both sequencing directions and independent PCR reaction. The three *TMPRSS3* missense mutations can be divided into three location groups: LDLRA domain mutant (p.R80H), SRCR domain mutant (p.L184S), and serine protease domain (p.A418V) (Figure 1D).

### 2.2. Analysis of Amino Acid Sequence Homology

**Figure 2:**
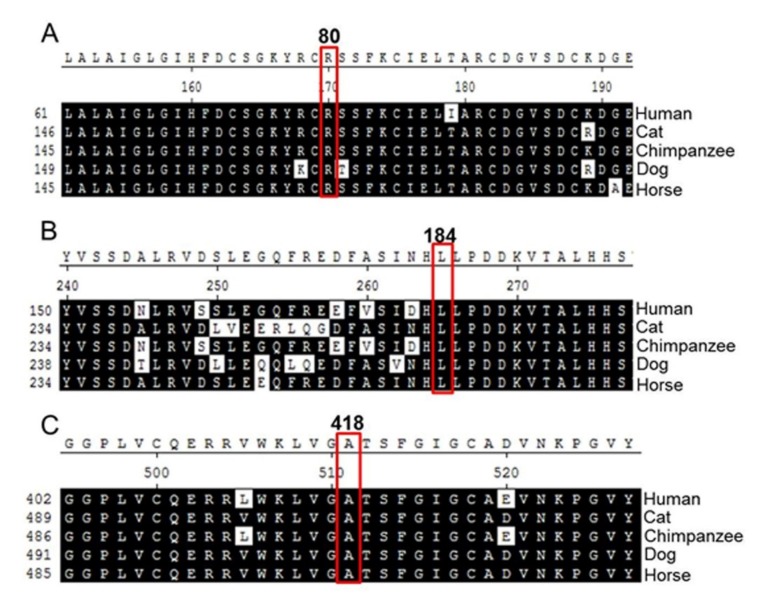
Alignment of the amino acid sequences of TMPRSS3 proteins of various species. The (**A**) p.Arg80, (**B**) p.Leu184, and (**C**) p.Ala418 residues, which were variants in this study, are indicated in bold. Arg (R) at codon 80, Leu (L) at codon 184, and Ala (A) at codon 418 are identical among all species compared.

In order to understand the effects of the p.R80H, p.L184S, and p.A418V missense variants, we examined the amino acid sequences of TMPRSS3 among various species using Biology WorkBench CLUSTAL W (1.81) Multiple Sequence Alignments (http://workbench.sdsc.edu/, San Diego Supercomputer Center). The comparison revealed that the amino acid sequences of codons p.R80H, p.L184S, and p.A418V were identical among the species examined (Figure 2). Therefore, we suggest that p.R80H, p.L184S, and p.A418V are specific to TMPRSS3 and likely play critical roles in its function.

### 2.3. Functional Study of TMPRSS3 Missense Mutation by sGASP

**Figure 3:**
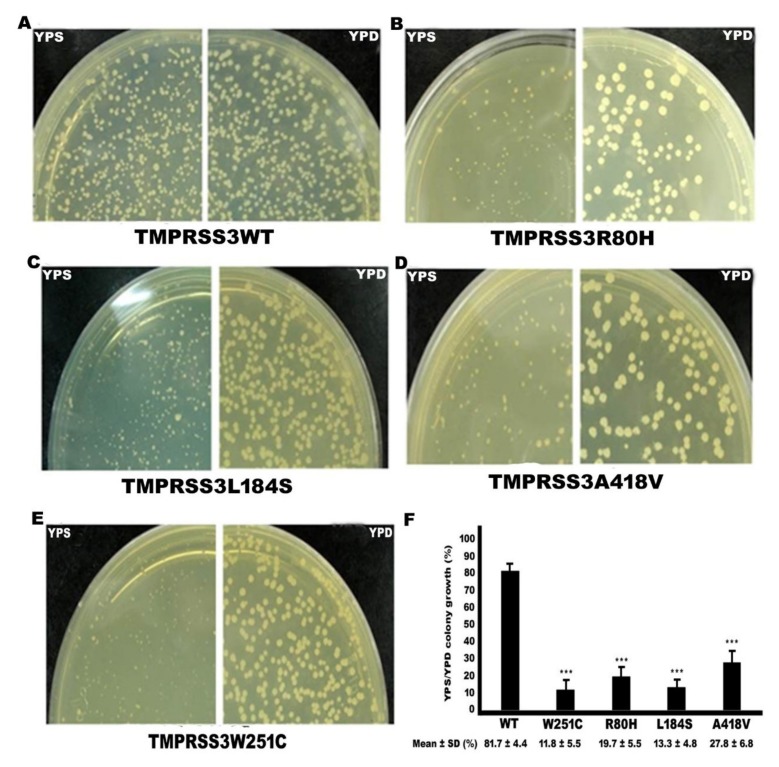
Assay of the catalytic activity of TMPRSS3 by site-specific proteolysis (sGASP). Transformants expressing both the STE13-substrate-invertase fusion protein and wild type (WT) or variants of the STE13-TMPRSS3 protein, including (**A**) TMPRSS3WT, (**B**) TMPRSS3R80H, (**C**) TMPRSS3L184S, (**D**) TMPRSS3A418V, and (**E**) TMPRSS3W251C, were plated on non-selective (glucose-YPD (yeast extract peptone dextrose)) and selective (sucrose-YPS (yeast extract peptone sucrose)) plates. TMPRSS3W251C mutant was as a positive control. (**F**) Quantitative assay of growth rate of yeast colony on sucrose plates. They were calculated by dividing the number of colony on sucrose plates by the number of colony on glucose plates (*n* = 5). ***: Asterisks denote the significant *p*-value <0.001.; STE13: Ste13p (Gene ID: 854394).

We determined whether the three missense mutations affect the proteolytic activity of TMPRSS3 by sGASP, which is effective for the study of secretory proteases [14,15]. The previously reported [15] pathogenic missense mutation p.W251C, which is located in the serine protease domain of TMPRSS3, was used as a positive control in the study (Figure 1D). The results of the sGASP analysis are shown in Figure 3. Our results indicate that the substrate sequence was cleavaged in the TMPRSS3WT co-expression condition, as shown by the growth of transformants on YPS plates (Figure 3A). In contrast, the three missense mutations, these being p.R80H, p.L184S, and p.A418V, had their activity significantly abolished, as evaluated by growth failure on YPS plates (Figure 3B–D). The pathogenic missense mutation, p.W251C (positive control), was significantly defective in protease activity (Figure 3E). Quantitative assays of yeast colonies on YPS plates after transformation of the mutations and wild type *TMPRSS3* gene determined the yeast growth rates were 19.4% (p.R80H), 13.2% (p.L184S), and 27.6% (p.A418V) after transformation of the three mutations of (Figure 3F). Our data indicate that the three missense mutations of TMPRSS3-transformed yeast growth rate were very few in number compared to TMPRSS3WT on YPS plate. Therefore, the pathogenesis of the three missense mutations were strongly correlated with the defective proteolytic activity of TMPRSS3.

### 2.4. Functional Study of TMPRSS3 Missense Mutation by Electrophysiological Measurements in Xenopus Oocytes

**Figure 4:**
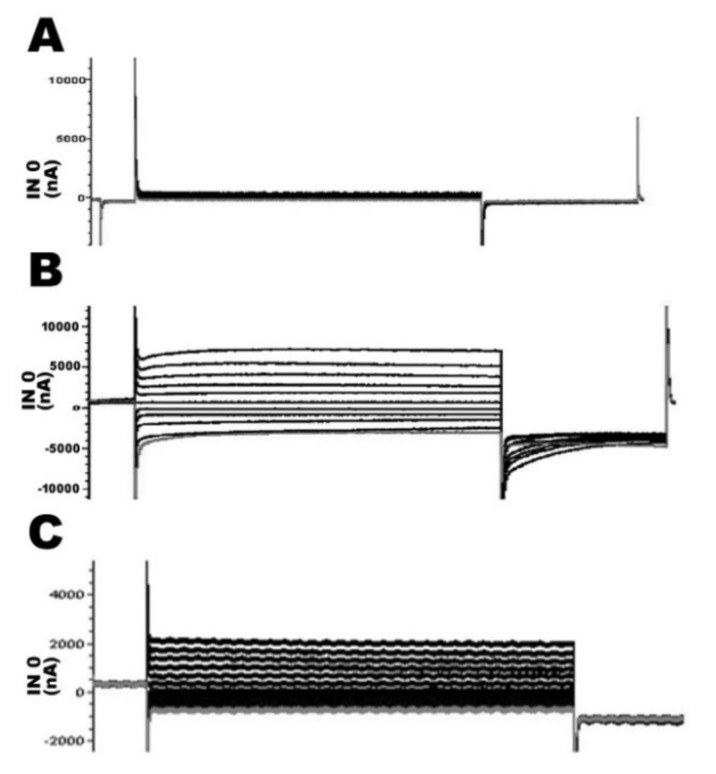
Assay of the Na^+^ currents of TMPRSS3 by electrophysiological measurements in Xenopus oocytes. The amiloride-sensitive current (INa) was measured in the presence of 120 μM of Na^+^ in Frog Ringer with 5 μM amiloride hydrochloride at a holding potential of 0 mV. (A) Due to the absence of any capped RNA (cRNA) as a negative control, the Na+ current was not produced and measured. (B) Co-injected cRNA of TMPRSS3WT and rat epithelial sodium channel (ENaC) α, β, and γ subunit into Xenopus oocytes, resulting in increased Na^+^ currents up to 5000–6000 nA (C) In contrast, the Na+ current was decreased to about 2000 nA in the amiloride hydrochloride treatment.

**Figure 5:**
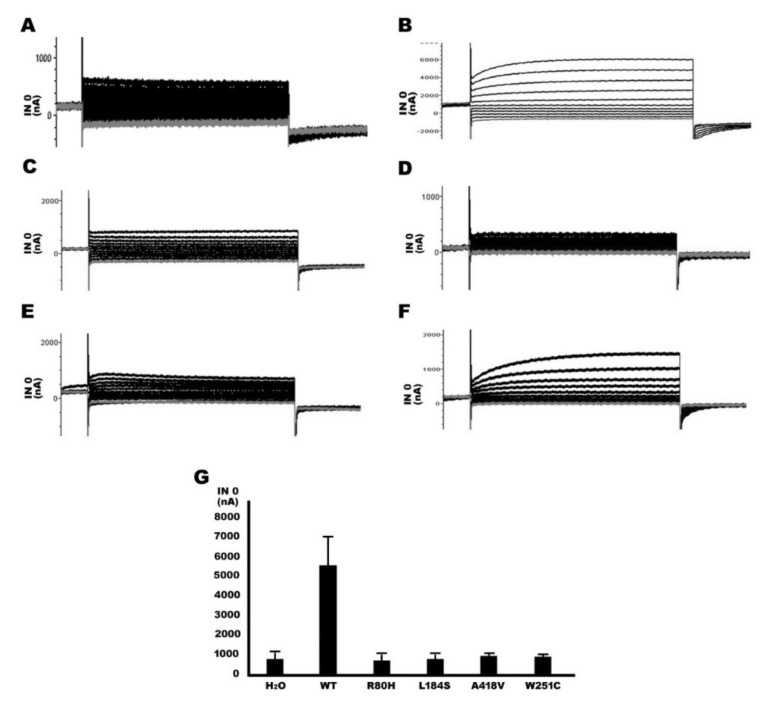
Functional expression of TMPRSS3 wild-type and mutant in Xenopus oocytes. Oocytes were injected with rat ENaC subunit in the presence of either (**A**) water (H_2_O), (**B**) TMPRSS3WT, and TMPRSS3 missense mutant, including (**C**) TMPRSS3R80H, (**D**) TMPRSS3L184S, (**E**) TMPRSS3A418V, and (**F**) TMPRSS3W251C. Water was used as a negative control. (**G**) All of the four missense mutant Na^+^ currents were similar to water, wherein there was no evident Na+ current and under 1000 nA in the quantitative analysis. In contrast, oocytes with the TMPRSS3WT emerging Na+ current were 5550 ± 1463 nA. (*n* ≥ 10).

**Figure 6:**
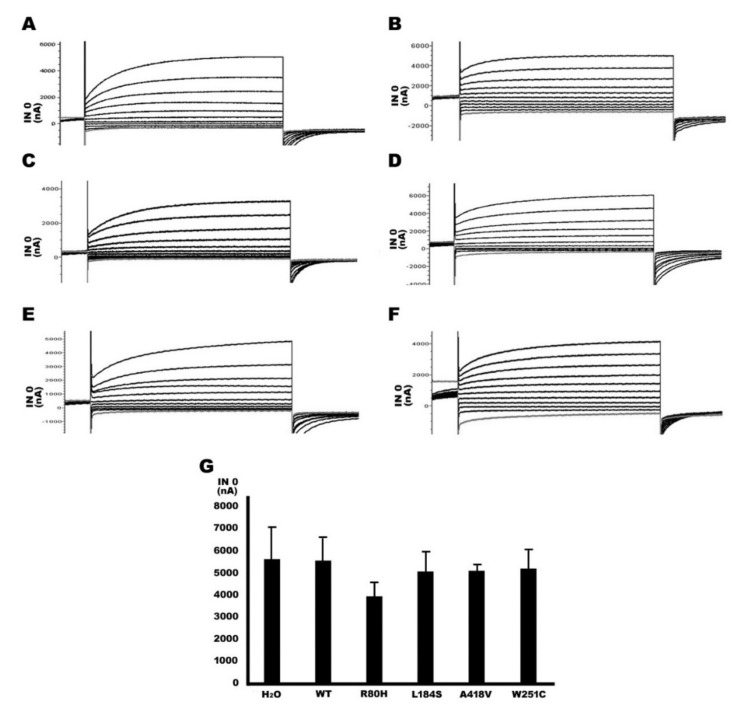
Serine protease trypsin can activate ENaC to produce Na+ current in Xenopus oocytes. The three rat ENaC subunits and one of water, WT, or mutant TMPRSS3 were co-injected into Xenopus oocytes. After injection, the oocytes were treated with trypsin and then Na+ currents were recorded. (**A**) After trypsin treatment, the ENaC was significant activated, producing Na+ currents in the water (H_2_O) group. (**B**) In the TMPRSS3WT group, whether there was a trypsin treatment or not did not affect the ENaC activation and the Na+ currents. (**C**–**F**) All of the four missense mutation groups were very similar to the water group, and ENaC could be activated, increasing the Na+ currents. (**G**) In the quantitative analysis, 10 Xenopus oocytes were treated by trypsin and measured by ENaC-mediated INa in each group. (*n* ≥10).

Apart from the sGASP analysis of yeast, we reconfirmed the functional effects of the TMPRSS3 mutation by electrophysiological measurements using Xenopus oocytes. Our results indicate that if Xenopus oocytes were not injected any capped RNA (cRNA) of rat ENaC α, β, and γ subunits and TMPRSS3WT, the Na+ current was not produced and measured (Figure 4A). In contrast, the four cRNA, including rat ENaC α, β, and γ subunits and TMPRSS3WT, were co-injected into Xenopus oocytes; the resulting proteolytic processing of TMPRSS3WT was associated with increased ENaC-mediated currents of up to 5000–6000 nanoampere (nA) (Figure 4B). An ENaC channel antagonist drug, amiloride, was used to verify the function of TMPRSS3WT; the results showed the current was decreased to about 2000 nA (Figure 4C). Furthermore, the oocytes injected only with the three rENaC subunits’ cRNA demonstrated no significant Na^+^ current compared to a co-expression of the TMPRSS3WT cRNA with the three rENaC subunit’s cRNA (Figure 5A vs. Figure 5B). In contrast, the three missense mutations (i.e., p.R80H, p.L184S, and p.A418V) reacted similarly to the positive control (p.W251C), by which the currents were decreased to about 1000–2000 nA (Figure 5C–F). The quantitative analysis is shown in Figure 5G and Table 4. Previous studies have shown that the serine protease trypsin I can activate ENaC [16,17]. In this study, our results indicate that trypsin was able to activate ENaC significantly in the absence of TMPRSS3 (Figure 6A). In the rENaC co-expressed with TMPRSS3WT, subsequent trypsin treatment had only a small additional effect (Figure 6B). These results showed a non-additive effect and the same signaling pathway between trypsin and TMPRSS3. By contrast, for the rENaC with TMPRSS3 mutant co-expression, INa currents were significantly enhanced by subsequent trypsin treatment (Figure 6C–F). The quantitative analysis is shown in Figure 6G and Table 3. According to the above results, we can conclude that the p.R80H, p.L184S, and p.A418V missense mutations impair TMPRSS3 proteolytic activation, thereby resulting in an inactive protein.

### 2.5. Functional Study of TMPRSS3 Heterozygous Missense Mutation by sGASP and Electrophysiological Measurements in Xenopus Oocytes

**Figure 7:**
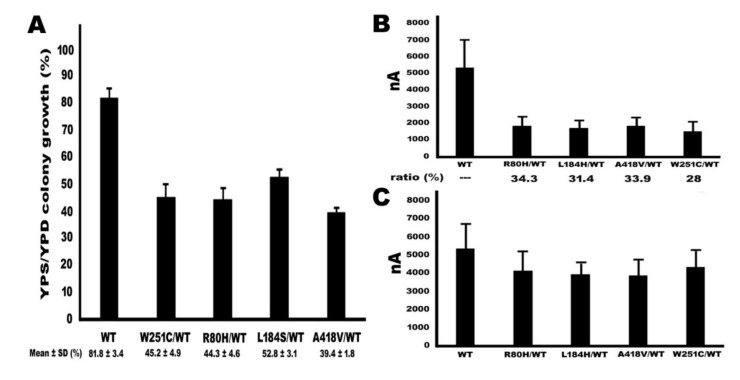
Analysis of the effects of the TMPRSS3 mutant proteins on TMPRSS3WT by co-expression studies in the sGASP system and Xenopus oocytes. (**A**) Yeast (KSY01 strain) was transformed with substrate and equal amounts expression plasmids of TMPRSS3WT and TMPRSS3 mutants, as indicated and plated on YPD and YPS plates. Quantitative assay of growth rate was calculated by dividing the number of colony on YPS plates by the number of colony on YPD plates. All values are expressed as the mean ± SD of duplicate determinations (*n* = 5). (**B**) Rat ENaC subunit and equal amounts of TMPRSS3WT and TMPRSS3 mutant cRNA co-injected into Xenopus oocytes for electrophysiological ratios were calculated (*n* ≥ 10). These results indicated that the Na+ currents were under 2000 nA in the four heterozygous mutations group. Simultaneously, the ratios of Na+ currents were calculated by dividing the currents of TMPRSS3 mutant by the currents of TMPRSS3WT. All of the four heterozygous mutation ratios were less than 40% function of TMPRSS3WT. (**C**) After trypsin treatment, the ENaC was significant activated, producing Na+ currents in the heterozygous mutation type.

In this study, our results found that the p.R80H, p.L184S, and p.A418V missense mutations of TMPRSS3 were heterozygous mutations in a NSHL patient. Therefore, co-expression studies were performed to examine the effects of the mutant proteins on TMPRSS3WT using sGASP analysis yeast and the electrophysiological measurements of Xenopus oocytes. To simulate the heterozygous genotype, co-transformants expressing three plasmids of a STE13-substrate-invertase fusion protein and equal amounts of STE13-TMPRSS3WT and TMPRSS3 mutation plasmids were plated on YPD and YPS plates (Figure 7A). Furthermore, we co-injected equal amounts of WT and mutant plasmids into Xenopus oocytes for electrophysiological evaluation (Figure 7B and Table 3). Simultaneously, we found a similar result to homozygous mutation, which is in the fact that the Na^+^ currents can increase by trypsin treatment in the heterozygous mutations type (Figure 7C and Table 3). These results of the above two experiments indicated that although heterozygous mutations have increased functional of protease and ENaC activity compared to homozygous mutations, their functions are still significantly reduced and less than 50% residual function compared to those of WT.

## 3. Discussion

TMPRSSs, which are involved in various physiological and pathological processes, are an emerging class of proteolytic enzyme [18,19]. Previous studies show that TMPRSS3 is a member of the TMPRSS family and causes autosomal recessive nonsyndromic hearing loss (ARNSHL) when its proteolytic ability is inactivated by 31 pathogenic mutations [7,12,13,15,20,21,22,23,24,25,26,27,28,29,30]. In contrast, our studies did not detect any of the aforementioned pathogenic variants and homozygous mutations in the *TMPRSS3* gene, whereas we identified three novel heterozygous missense mutations, c.239 G>A (p.R80H), c.551 T>C (p.L184S), and c.1253 C>T (p.A418V), in patients with NSHL. Moreover, those three missense *TMPRSS3* mutations were not detected in the 120 normal hearing individuals. We suggest that differences in mutations of *TMPRSS3* are due possibly to racial variation. In the patients carrying a *TMPRSS3* mutation, we were unable to identify any mutations in members of the connexin (Cx) gene family in our previous studies [31,32]. However, we cannot rule out the possibility that an interaction of *TMPRSS3* mutants with a second gene, at the same or at another locus, exerts a dominant effect. It is also possible that these nucleotide changes represent uncommon polymorphisms not associated with the disorder phenotype in these individuals. The exact relationship remains to be assessed in the further. Whole exome analysis is a good method to solve the problem.

Our results indicate that p.R80, p.L184, and p.A418 are highly conserved residues in the TMPRSS3 protein, suggesting that the site of the three amino acids plays an important functional role specific to the TMPRSS3 protein (Figure 2). The conversion of the three missense mutations with side chains of different sizes and properties may alter proximal intramolecular binding and may lead to the loss of the TMPRSS3 protein’s function. The TMPRSS3 protein’s major structure possesses three functional domains, the LDLRA, SRCR, and serine protease domains [8]. According to previous studies, when the structural changes of SRCR and LDRRA have caused incorrect folding in the SRCR and LDRRA regions, this was because the target protein was unable to recognize the binding site [15]. In addition, the serine protease domain has shown that an important ability of TMPRSS3 is to activate the ENaC sodium channel, along with its catalytic activity. The amiloride-sensitive ENaC, which is expressed in many Na+-reabsorbing tissues, such as the inner ear, is regulated by membrane-bound channel activating serine protease (CAPs), and is involved in the regulation of the sodium concentration in the endolymph [15,20].

Previous studies have demonstrated that the mutations the three domains produce may lead to a loss of function and cause HL, for example, p.D103G in the LDLRA domain, p.C194F in the SRCR domain, and p.W251C in the serine protease domain [20,22]. In this study, we found that three missense mutations, p.R80H, p.L184S, and p.A418V, were located in the LDRRA, SRCR, and serine protease domains, respectively (Figure 1D). In an sGASP functional study of the TMPRSS3 protein, the three missense mutations (p.R80H, p.L184S, p.A418V) all significantly diminished the protein’s activity (Figure 3). In addition, the three missense mutations all failed to undergo proteolytic cleavage and to activate ENaC in the Xenopus oocyte expression system (Figure 5 and Figure 6). Therefore, we suggest that the three missense mutations could be pathogenic in certain circumstances and could thus worsen the situation and eventually lead to pathogenesis.

All previous studies indicate that a recessive mutation form in the *TMPRSS3* gene is associated with NSHL [12]. Nevertheless, we only found heterozygous mutations forming in the *TMPRSS3* gene and a low incidence rate in Taiwanese patients with NSHL. Clinical penetrance depended in part on function of the mutation in question. For specific disease, some causal mutations may show 100% penetrance, whereas other mutations in the same gene show reduced penetrance or even very low penetrance. Thus, penetrance is the likelihood of any phenotypic effect caused by a particular genotype [33]. The unlinked modifier genes, epigenetic changes, or environmental factors are possible modifiers of penetrance [34]. In addition, the two alleles of the disease gene may be expressed at different levels in autosomal dominant conditions; this inconsistency can preference either the mutant or the wild-type allele and hence may influence clinical penetrance in either direction [35]. Therefore, clinic penetrance may be explained by this phenomenon, which is heterozygous *TMPRSS3* mutation’s association with NSHL in our study. Moreover, we also found that heterozygous mutations increased their function compared to homozygous mutations, but their functions were still significantly reduced and comprised less than 50% residual function compared to WT in the functional studies of heterozygous mutations (Figure 7). Therefore, our data seem to indicate that the p.R80H, p.L184S, and p.A418V of TMPRSS3 heterozygous mutations may be associated with NSHL. However, we cannot rule out the reason for the decrease in TMPRSS3 activity function may be due to only halving the WT plasmid. In addition, we cannot rule out the possibility that the nucleotide changes represent a rare or uncommon polymorphism not associated with the disease phenotype in the patients. Therefore, the exact relationship remains to be assessed in further research.

The involvement of TMPRSS3 in HL suggests the possible implication of other TMPRSS members, as previously shown for members of the myosin superfamily [36,37,38]. Previous studies have indicated that another member of this subfamily, TMPRSS1, can be important for normal hearing [39]; TMPRSS1-null cochleae displayed abnormal tectorial membrane development, a reduction in nerve fiber compaction, and decreased expression of the myelin proteins. In addition, TMPRSS2, -5, and -10 were found to be expressed in inner ear tissues and were mapped within the HL loci PKSR7 (Pakistani family), autosomal recessive nonsyndromic deafness-24 (DFNB24), and autosomal recessive nonsyndromic deafness-25 (DFNB25), respectively [11,19]. Therefore, we cannot rule out the possibility that there are other genes whose mutations are associated with the NSHL in these individuals. The exact relationship remains to be determined.

## 4. Materials and Methods

### 4.1. Research Subjects

We screened a total of 230 unrelated patients (age range, 4–18 years) with severe to profound NSHL. Pure tone audiometry (PTA) was performed on all patients. The frequency range of HL was 250–8000 Hz, with a mean threshold of more than 70 dB in both right and left ears. Individuals with syndromic HL or environment-related HL, as determined by otorhinolaryngologists, were excluded from the study. We also randomly selected 120 healthy individuals (age range of 17–22 years) with normal hearing (mean threshold less than 10 dB) as the control group. All procedures were approved (CS03045) by the Chung Shan Medical University Hospital Institutional Review Board. Informed consent to use genetic material was obtained from the parents of all participants in this study.

### 4.2. Detection of TMPRSS3 Gene Mutations

The genomic DNA was extracted from peripheral blood samples (3 mL) taken from each individual. The DNA was purified using a Gentra DNA Blood Kit (Qiagen, Hilden, Germany) according to the manufacturer’s instructions. The quality and quantity of the purified genomic DNA were determined by gel electrophoresis and spectrophotometry. Variant screenings were performed by direct sequencing of the selected exons and flanking intronic sequences of the *TMPRSS3* gene. DNA fragments containing *TMPRSS3* exons 1 to 13 were first amplified by polymerase chain reaction (PCR) using genomic DNA samples with intragenic primers (Table 4). In brief, the PCR was carried out in a total volume of 25 μL, containing 100 ng of genomic DNA, 200 mM deoxy-ribonucleoside triphosphate (dNTP), 0.25 units of proTaq DNA polymerase (Promega, Madison, Wisconsin, United States), and 200 mM intragenic primers. The PCR reaction conditions for different exons are shown in Table 4. The PCR products were then confirmed by electrophoresis and purified by a DNA extraction kit (Viogene, Sijhih, Taitung). The PCR products were then subjected to PCR-directed DNA sequencing using a DNA sequencing kit (Applied Biosystems Corporation, Foster City, CA, United States) and an Applied Biosystems model 3730 automated sequencer (Applied Biosystems Corporation, Foster City, CA, United States). Sequence data were compared with the published exons sequence of *TMPRSS3* (GeneBank Accession Nos. NC_000021).

### 4.3. Molecular Cloning and Construct of Wild-Type (WT) and Mutant TMPRSS3 Expression Plasmids for Functional Study

For a yeast base assay, the wild type of STE13-TMPRSS3 (TMPRSS3WT) plasmid was provided by Professor Park’s laboratory in South Korea [14]. For the Xenopus oocytes study, the open reading frames (ORFs) of *TMPRSS3* were obtained from human colon tissue by RT-PCR. A PCR was carried out with the following oligonucleotide primers: the forward primer was TMPRSS3-NcoI (restriction enzyme), 5’AATCCATGGGGGAAAATGATCCGCC3’, and corresponded to nucleotides 1–20 of the human TMPRSS3 coding region; the reverse primer was TMPRSS3R-Xho1(restriction enzyme), 5’ATTCTCGAGTCAGGTTTTTAGGTCTCTCTCC3’, and corresponded to nucleotides 1343–1365 of the human TMPRSS3 coding region (the enzyme site nucleotide is underlined). The PCR DNA product (1365 bp) of the human *TMPRSS3* coding region was cloned into a pGEM®-T vector using a pGEM-TA clone kit (Promega, Madison, WI, USA) according to the manufacturer’s recommendations. Next, TMPRSS3WT was subcloned into the expression vector pTLN, which is for Xenopus oocytes expression vector with SP6 promoter and is described in a previous study [40], using NcoI and Xho1 restriction enzymes (New England Biolabs, Ipswich, Massachusetts, United States).

Mutant *TMPRSS3* expression plasmids were obtained from TMPRSS3WT as a template using the Stratagene Quickchange site-directed mutagenesis kit according to the manufacturer’s recommendations (Stratagene, La Jolla, CA, USA). The oligonucleotide primers for preparing the mutant TMPRSS3 expression plasmids are summarized in Table 1. The α, β, and γ subunits of rat ENaC, which can form a sodium ion channel in the Xenopus oocytes, were cloned for functional study in the Xenopus oocytes system. Previously, the three subunits were reported to produce high expression in the kidney of a rat [41]. Therefore, the ORFs of α, β, and γ subunits were obtained from the kidney tissue of a rat by RT-PCR. A PCR was carried out with the oligonucleotide primers shown in Table 1. The RT-PCR products were cloned into an pGEM-T Easy Vector according to the manufacturer’s recommendations (Promega, Madison WI). The three subunits were then subcloned into the pTLN vector using Hind III and Sac II restriction enzymes (New England Biolabs). All constructs were confirmed by the dideoxy DNA sequencing method using an ABI Prism 3730 Genetic Analyzer (Applied Biosystems Corporation, Foster City, CA).

### 4.4. sGASP Assay (Secretory Genetic Assay of Site-Specific Proteolysis)

The yeast strain KSY01, a kex2 mutant strain, (MATa leu2 ura3 his3 trp1 lys2 suc2-D9 kex2::HIS3) for the sGASP system was provided by Professor Park’s laboratory in South Korea [14]. The procedure was performed according to the related literature [15]. Both the STE13-substrate-invertase fusion protein and STE13-TMPRSS3 (wild type or mutants) were introduced into yeast strain KSY01 by lithium acetate transformation. After transformation, the yeast was plated on non-selective (yeast extract peptone dextrose; YPD) and selective (yeast extract peptone sucrose; YPS) plates. Colonies usually appeared in 7 days at 30 °C. They were calculated by dividing the number of colonies on the YPS plates by the number of colonies on the glucose plates. The rations are plating efficiencies, which represent relative proteolytic activity.

### 4.5. cRNA Preparation for This Study

The preparation method follows the one reported by Kubisch et al. (1998) [40]. The pTLN plasmids within WT or mutant TMPRSS or rat α-, β-, and γ- ENaC subunits were linearized with HpaI (New England Biolabs, Ipswich, Massachusetts, United States). For synthesizing cRNA(Capped RNA), the mMessage mMachine SP6 transcription kit (Ambion, Austin, TX, United States) was used according to the manufacturer’s recommendations. All synthesized cRNA was stored in a −80 °C refrigerator for subsequent experiments.

### 4.6. Electrophysiological Measurements in Xenopus Oocytes

The method of electrophysiological measurements is a slightly modified version of the one used by Guipponi et al. (2002) [20]. Xenopus oocytes were isolated from the abdomen of *Xenopus laevis* (Noerdhoek, South Africa). Stage V or VI healthy oocytes were then microinjected with 0.25 ng of each cRNA coding for the rat α-, β-, and γ-ENaC subunits in the presence or absence of 5 ng of WT or mutant TMPRSS3 cRNA in a total volume of 50 nL using a Drummond nanoject apparatus. Forty-eight hours after injection, the oocytes were analyzed for Na ion transport using a two-electrode voltage clamp technique. The amiloride-sensitive current (INa) was measured in the presence of 120 μM of Na^+^ in Frog Ringer solution with 5 μM amiloride at a holding potential of 0 mV. To confirm these results, a prototypical serine protease trypsin I known to activate ENaC was used in the study. The oocytes were perfused with 2 mg/mL of trypsin for 2–3 min, and the INa was re-measured. Three series of experiments were performed, each with five oocytes per condition. All results are reported as means ± SEM (standard error of the mean). In experiments where oocytes were perfused with trypsin, *n* represents the number of experiments performed. Amiloride hydrochloride and trypsin I was purchased from Sigma-Aldrich (Taufkirchen, Germany), and the amiloride was prepared as a 10 mM aqueous stock solution. All animal care and experimental procedures were performed according to the guidelines of the Animal Care Committee of the Chung Shan Medical University.

## Figures and Tables

**Table 1:** Polymorphisms of *TMPRSS3* gene in the study.

Polymorphism				Allele Frequency (%)		
TMPRSS3 Protein Domain	Region	Genotype	Amino Acid Change	Hearing Loss	Control	*p*-Value *
				(*n* = 460)	(*n* = 240)	
5′-UTR		−78T>A	-----	T: 0.997 A: 0.003	T:0.996 A:0.004	0.742658
		−36G>C	-----	G:0.995 A:0.005	G:0.996 A: 0.004	0.848021
TM	Exon 3	157G>A	V53I	G:0.750 A:0.250	G:0.867 A:0.133	0.000455 ^&^
	Intron 5	447-13A>G	-----	A:0.421 G:0.579	A:0.582 G:0.408	0.000035 ^&^
SRCR	Exon 6	453G>A	V151V	G:0.608 A:0.392	G:0.754 A:0.246	0.000172 ^&^
	Intron 7	617-4 ins AT	-----	--:0.663 AT:0.337	--:0.754 AT:0.246	0.016173 ^&^
Serine protease	Exon 8	681G>A	Q227Q	G:0.989 A:0.011	G:0.987 A:0.013	0.820762
		757A>G	I253V	A:0.626 G:0.374	A:0.742 G:0.258	0.002906 ^&^
		763G>T	A255A	G:1.000 T:0.000	G:0.996 T:0.004	#
	Exon9	945G>A	T315T	G:1.000 A:0.000	G:0.996 A:0.004	#
	Intron9	952 + 18T>C	-----	T:1.000 C:0.000	T:0.996 C:0.004	#
	Intron10	1048 + 56C>T	-----	C:0.982 T:0.018	C:0.987 T:0.013	0.568631
	Exon11	1121A>G	D374G	A:0.992 G:0.008	A:0.992 G:0.008	0.952574
		1122C>T	D374D	C:0.992 T:0.008	C:0.996 T:0.004	0.572243
	Exon12	1335C>T	H445H	C:0.976 T:0.024	C:0.987 T:0.013	0.324825
3′-UTR	Exon 13	1367G>A	-----	G:0.242 A:0.758	G:0.517 A:0.483	<0.000001 ^&^
		1399 + 19A>G	-----	A:0.995 G:0.005	A:0.992 G:0.008	0.641868

* Chi-square test was significant. # The variant only in control group. ----- no encode region for amino acid. ^&^ Between the patients and the controls was significant (*p* < 0.05). -- no nucleotide.

**Table 2:** Proportion of mutations of *TMPRSS3* gene in 230 patients with nonsyndromic hearing loss (NSHL).

Location	Nucleotide Change	Domain	Amino Acid Change	Predicted Effect	Individual Found	Percentage (%)
Exon1	−75 A>G/wt	---	X	N	1	0.43%
Exon2	239G>A/WT	LDLRA	R80H	Missense mutation	1	0.43
Intron3	205+38 C>T/WT	---	X	Splicing	5	2.17%
Exon6	551T>C/WT	SRCR	L184S	Missense mutation	1	0.43%
Exon8	621T>C/WT	Serine protease	C207C	Neutral (or silent) mutation	1	0.43%
Exon9	933C>T/WT	Serine protease	A311A	Neutral (or silent) mutation	1	0.43%
Intron11	1194+15C>A/WT	---	X	Splicing	1	0.43%
Exon12	1253 C>T/WT	Serine protease	A418V	Missense mutation	1	0.43%
Exon12	1269 C>T/WT	Serine protease	I423I	Neutral (or silent) mutation	1	0.43%
Intron12	1347+11 C>T/WT	---	X	Splicing	1	0.43%
	Total				14	6.09%

---: Variants located in non-translation region or intron; X: amino acid no changed; N: unpredictable

**Table 3:** Electrophysiological measurements in Xenopus oocytes.

Genotype (Homozygous)	*No Trypsin (INa Current, nA)	*Trypsin (INa Current, nA)
Water (H_2_O)	786 ± 345	5607 ± 1456
TMPRSS3WT/TMPRSS3WT	5550 ± 1463	5551 ± 1047
TMPRSS3R80H/TMPRSS3R80H	702 ± 374	3936 ± 615
TMPRSS3L184S/TMPRSS3L184S	791 ± 261	5060 ± 889
TMPRSS3A418V/TMPRSS3A418V	927 ± 63	5084 ± 292
TMPRSS3W251C/TMPRSS3W251C	888 ± 89	5181 ± 889
**Genotype (heterozygous)**	*No Trypsin (INa current, nA)	*Trypsin (INa current, nA)
TMPRSS3WT/ TMPRSS3WT	5337 ± 1662	5351 ± 1368
TMPRSS3R80H/ TMPRSS3WT	1828 ± 524	4130 ± 1075
TMPRSS3L184S/ TMPRSS3WT	1674 ± 467	3910 ± 701
TMPRSS3A418V/TMPRSS3WT	1811 ± 509	3848 ± 717
TMPRSS3W251C/TMPRSS3WT	1493 ± 606	4320 ± 954

- * means ± SEM (standard error of the mean) (*n* ≥10); nA: nanoampere.

**Table 4:** Primers of transmembrane protease serine 3 (*TMPRSS3*) gene for screening in hearing loss and normal hearing individuals.

Region	Primer Sequence (Oligonucleotide)	Annealing, for PCR Temperature (°C)	Product Size
Exon 1	F: 5′-CTCTGGTCTCCTTGGCATTGTG-3 R: 5′-CTCTCAAAGCCCTTTCCATTGC-3′	61	387 bps
Exon 2	F: 5′-GATGCACCTGATGCTACAAG-3′ R: 5′-GGACAGTCAGTCACATTGGTC-3′	55	286 bps
Exon 3	F: 5′-GGACTGAACTAGAGAATGTGCC-3′ R: 5′-GACAAAGCCATGAGCATGGC-3′	63	510 bps
Exon 4	F: 5′-AGGGGGACAGTTGTTAGTGTTGC-3′ R: 5′-TACAGATGGGAAGGGTCAGGGTTG-3′	62	261 bps
Exon 5	F: 5′-TGTGGAGAAACCCCTGCCTATG-3′ R: 5′-GATGTGAGGATGTAATCTGAGAGCG-3′	68	323 bps
Exon 6	F: 5′-GACTCGCACATCGGTTGAATG-3′ R: 5′-ATACTCCCTCAGGTTCTCACACCC-3′	64	387 bps
Exon 7	F: 5′-GTGTGACCTCATCCTCATGG-3′ R: 5′-CTCTGAGGGCAAGGAGATAG-3′	64	293 bps
Exon 8	F: 5′-TAGAGCTGCTGTGAGCTCTG-3′ R: 5′-AGACTCCTCTCCAACTGTAC-3′	62	438 bps
Exon 9	F: 5′-GGACCACATCTTGCCTGATAACC-3′ R: 5′-AAAGCACACAGCCCACGAAG-3′	62	694 bps
Exon 10	F: 5′-CTCCTGCTGTGAGCTGATCG-3′ R: 5′-CGAGCAGCTGACATGCACTC-3′	61	393 bps
Exon 11	F: 5′-GTCTCAGCATGCGCCTTCTG-3′ R: 5′-CCCACGCAGAGCCAGATCAC-3′	65	407 bps
Exon 12	F: 5′-TGGGTCATCCATTGGGACATC-3′ R: 5′-TCTCTGTTTTCAGCACAAGCGTC-3′	64	460 bps
Exon 13	F: 5′-TACGGAAGTGACGGAACTGTCG-3′ R: 5′-CTTGAAGGTTGTGCTGGAATCAG-3′	64	442 bps

F: forward; R: reverse; bp: base pair.

## References

[1] Apps S.A., Rankin W., Kurmis A.P. (2007). Connexin 26 mutations in autosomal recessive deafness disorders: A review. Int. J. Audiol..

[2] Morton N.E. (1991). Genetic epidemiogy of hearing impairment. Ann. NY Acad. Sci..

[3] Pallares-Ruiz N., Blanchet P., Mondain M., Claustres M., Roux A.F. (2002). A large deletion including most of GJB6 in recessive nonsyndromic deafness: A digenic effect?. Eur. J. Hum. Genet..

[4] Jung J., Lee J.S., Cho K.J., Yu S., Yoon J.-H., Gee H.Y., Choi J.Y. (2017). Genetic Predisposition to Sporadic Congenital Hearing Loss in a Pediatric Population. Sci. Rep..

[5] Bosanquet D., Williams N., Lewis M. (2010). Acquired small bowel diverticular disease: A review. Br. J. Hosp. Med..

[6] Szabo R., Wu Q., Dickson R.B., Netzel-Arnett S., Antalis T.M., Bugge T.H. (2003). Type II transmembrane serine proteases. Thromb. Haemost..

[7] Scott H.S., Kudoh J., Wattenhofer M., Shibuya K., Berry A., Chrast R., Guipponi M., Wang J., Kawasaki K., Asakawa S. (2001). Insertion of beta-satellite repeats identifies a transmembrane protease causing both congenital and childhood onset autosomal recessive deafness. Nat. Genet..

[8] Hooper J.D., Clements J.A., Quigley J.P., Antalis T.M. (2001). Type II transmembrane serine proteases Insights into an emerging class of cell surface proteolytic enzymes. J. Biol. Chem..

[9] Bonné-Tamir B., DeStefano A.L., Briggs C.E., Adair R., Franklyn B., Weiss S., Korostishevsky M., Frydman M., Baldwin C.T., Farrer L.A. (1996). Linkage of congenital recessive deafness (gene DFNB10) to chromosome 21q22.3. Am. J. Hum. Genet..

[10] Veske A., Oehlmann R., Younus F., Mohyuddin A., Müller-Myhsok B., Mehdi S.Q., Gal A. (1996). Autosomal Recessive Non-Syndromic Deafness Locus (DFNB8) Maps on Chromosome 21Q22 in a Large Consanguineous Kindred from Pakistan. Hum. Mol. Genet..

[11] Guipponi M., Toh M.-Y., Tan J., Park D., Hanson K., Ballana E., Kwong D., Cannon P.Z., Wu Q., Gout A. (2007). An integrated genetic and functional analysis of the role of type II transmembrane serine proteases (TMPRSSs) in hearing loss. Hum. Mutat..

[12] Gao X., Huang S.-S., Yuan Y.-Y., Xu J.-C., Gu P., Bai D., Kang D.-Y., Han M.-Y., Wang G.-J., Zhang M.-G. (2017). Identification ofTMPRSS3as a Significant Contributor to Autosomal Recessive Hearing Loss in the Chinese Population. Neural Plast..

[13] Gao X., Yuan Y.-Y., Wang G.-J., Xu J.-C., Su Y., Lin X., Dai P. (2017). Novel Mutations and Mutation Combinations ofTMPRSS3Cause Various Phenotypes in One Chinese Family with Autosomal Recessive Hearing Impairment. BioMed Res. Int..

[14] Kim S.Y., Park D., Oh M., Sellamuthu S., Park W.J. (2002). Detection of site-specific proteolysis in secretory pathways. Biochem. Biophys. Res. Commun..

[15] Lee Y.J., Park D., Kim S.Y., Park W.J. (2003). Pathogenic mutations but not polymorphisms in congenital and childhood onset autosomal recessive deafness disrupt the proteolytic activity of TMPRSS3. J. Med Genet..

[16] Nesterov V., Dahlmann A., Bertog M., Korbmacher C. (2008). Trypsin can activate the epithelial sodium channel (ENaC) in microdissected mouse distal nephron. Am. J. Physiol. Physiol..

[17] Haerteis S., Krappitz A., Krappitz M., Murphy J.E., Bertog M., Krueger B., Nacken R., Chung H., Hollenberg M.D., Knecht W. (2014). Proteolytic Activation of the Human Epithelial Sodium Channel by Trypsin IV and Trypsin I Involves Distinct Cleavage Sites. J. Boil. Chem..

[18] Wu Q. (2003). Type II transmembrane serine proteases. Cur. Top. Develop. Biol..

[19] Bugge T.H., Antalis T.M., Wu Q. (2009). Type II Transmembrane Serine Proteases*. J. Boil. Chem..

[20] Guipponi M., Vuagniaux G., Wattenhofer M., Shibuya K., Vazquez M., Dougherty L., Scamuffa N., Guida E., Okui M., Rossier C. (2002). The transmembrane serine protease (TMPRSS3) mutated in deafness DFNB8/10 activates the epithelial sodium channel (ENaC) in vitro. Hum. Mol. Genet..

[21] Ben-Yosef T., Wattenhofer M., Riazuddin S., Ahmed Z.M., Scott H.S., Kudoh J., Shibuya K., Antonarakis S.E., Bonne-Tamir B., Radhakrishna U. (2001). Novel mutations of TMPRSS3 in four DFNB8/B10 families segregating congenital autosomal recessive deafness. J. Med. Genet..

[22] Masmoudi S., Antonarakis S.E., Schwede T., Ghorbel A.M., Gratri M., Pappasavas M.-P., Drira M., Elgaied-Boulila A., Wattenhofer M., Rossier C. (2001). Novel missense mutations of TMPRSS3 in two consanguineous Tunisian families with non-syndromic autosomal recessive deafness. Hum. Mutat..

[23] Wattenhofer M., Di Iorio M., Rabionet R., Dougherty L., Pampanos A., Schwede T., Montserrat-Sentís B., Arbones M., Iliades T., Pasquadibisceglie A. (2002). Mutations in the TMPRSS3 gene are a rare cause of childhood nonsyndromic deafness in Caucasian patients. J. Mol. Med..

[24] Wattenhofer M., Sahin-Calapoglu N., Andreasen D., Kalay E., Caylan R., Braillard B., Fowler-Jaeger N., Reymond A., Rossier B.C., Karaguzel A. (2005). A novel TMPRSS3 missense mutation in a DFNB8/10 family prevents proteolytic activation of the protein. Hum. Genet..

[25] Weegerink N.J., Schraders M., Oostrik J., Huygen P.L., Strom T.M., Granneman S., Pennings R.J., Venselaar H., Hoefsloot L.H., Elting M. (2011). Genotype phenotype correlation in DFNB8/10 families with TMPRSS3 mutations. J. Assoc. Res. Otolaryngol..

[26] Hutchin T., Coy N., Conlon H., Telford E., Bromelow K., Blaydon D., Taylor G., Coghill E., Brown S., Trembath R. (2005). Assessment of the genetic causes of recessive childhood non-syndromic deafness in the UK - implications for genetic testing. Clin. Genet..

[27] Battelino S., Klančar G., Kovač J., Battelino T., Podkrajsek K.T. (2015). TMPRSS3 mutations in autosomal recessive nonsyndromic hearing loss. Eur. Arch. Oto-Rhino-Laryngology.

[28] Lee J., Baek J.-I., Choi J.Y., Kim U.-K., Lee S.-H., Lee K.-Y. (2013). Genetic analysis of TMPRSS3 gene in the Korean population with autosomal recessive nonsyndromic hearing loss. Gene.

[29] Fan D., Zhu W., Li D., Ji D., Wang P. (2014). Identification of a Novel Homozygous Mutation, TMPRSS3: C.535G>A, in a Tibetan Family with Autosomal Recessive Non-Syndromic Hearing Loss. PLoS ONE.

[30] Guipponi M., Antonarakis S.E., Scott H.S. (2008). TMPRSS3, a type II transmembrane serine protease mutated in non-syndromic autosomal recessive deafness. Front. Biosci..

[31] Yang J.-J., Huang S.-H., Chou K.-H., Liao P.-J., Su C.-C., Li S.-Y. (2007). Identification of Mutations in Members of the Connexin Gene Family as a Cause of Nonsyndromic Deafness in Taiwan. Audiol. Neurotol..

[32] Yang J.-J., Wang W.-H., Lin Y.-C., Weng H.-H., Yang J.-T., Hwang C.-F., Wu C.-M., Li S.-Y. (2010). Prospective variants screening of connexin genes in children with hearing impairment: Genotype/phenotype correlation. Qual. Life Res..

[33] Cooper D.N., Krawczak M., Polychronakos C., Tyler-Smith C., Kehrer-Sawatzki H. (2013). Where genotype is not predictive of phenotype: Towards an understanding of the molecular basis of reduced penetrance in human inherited disease. Qual. Life Res..

[34] Zlotogora J. (2003). Jo Penetrance and expressivity in the molecular age. Genet. Med..

[35] De La Chapelle A. (2009). Genetic predisposition to human disease: Allele-specific expression and low-penetrance regulatory loci. Oncogene.

[36] Gibson F., Walsh J., Mburu P., Varela A., Brown K.A., Antonio M., Beisel K.W., Steel K.P., Brown S.D.M. (1995). A type VII myosin encoded by the mouse deafness gene shaker-1. Nature.

[37] Well D., Blanchard S., Kaplan J., Guilford P., Gibson F., Walsh J., Mburu P., Varela A., Levilliers J., Weston M.D. (1995). Defective myosin VIIA gene responsible for Usher syndrome type IB. Nature.

[38] Donaudy F., Ferrara A., Esposito L., Hertzano R., Ben-David O., Bell R.E., Melchionda S., Zelante L., Avraham K.B., Gasparini P. (2003). Multiple Mutations of MYO1A, a Cochlear-Expressed Gene, in Sensorineural Hearing Loss. Am. J. Hum. Genet..

[39] Guipponi M., Tan J., Cannon P.Z., Donley L., Crewther P., Clarke M., Wu Q., Shepherd R.K., Scott H.S. (2007). Mice Deficient for the Type II Transmembrane Serine Protease, TMPRSS1/hepsin, Exhibit Profound Hearing Loss. Am. J. Pathol..

[40] Kubisch C., Schroeder B.C., Friedrich T., Lutjohann B., El-Amraoui A., Marlin S., Petit C., Jentsch T.J. (1998). KCNQ4, a novel potassium channel expressed in sensory outer hair cells, is mutated in dominant deafness. Cell.

[41] Cecilia M.C., Laurent S., Gary B., Bernard T., Ivan G., Jran-Daniel H., Bernard C.R. (1994). Amiloride-sensitive epithelial Na+ channel is msde of three homologous subunits. Nature.

